# Fracture Hunting in Ruby-Throated Hummingbirds (*Archilochus colubris*): A Comparative Study of General Radiography, Dental Radiography, Micro-CT, and 3D Reconstructed Imaging

**DOI:** 10.3390/ani16010062

**Published:** 2025-12-25

**Authors:** Haerin Rhim, Kimberly L. Boykin, Zoey Lex, Katie Bakalis, Rachel Jania, Kassandra Wilson, Devin Osterhoudt, Mark A. Mitchell

**Affiliations:** 1 Department of Veterinary Clinical Sciences, School of Veterinary Medicine, Louisiana State University, Baton Rouge, LA 70803, USAmmitchell@lsu.edu (M.A.M.); 2 Department of Pathobiological Sciences, School of Veterinary Medicine, Louisiana State University, Baton Rouge, LA 70803, USA

**Keywords:** bird, hummingbird, radiography, computed tomography, 3D, fracture, wildlife

## Abstract

Diagnosing fractures in hummingbirds is challenging because of their small size. This study evaluated the diagnostic performance and inter-reviewer agreement of four imaging modalities—conventional radiography, dental radiography, micro-computed tomography (micro-CT), and three-dimensional (3D)-reconstructed images from micro-CT scans—for identifying fractures in 16 ruby-throated hummingbirds (*Archilochus colubris*) admitted to a wildlife hospital. Six independent reviewers, with or in training for a specialty in veterinary radiology or wildlife medicine, assessed randomized image sets. Gross dissection of the carcasses using dermestid beetle larvae established the gold standard. Diagnostic performance metrics—sensitivity, specificity, predictive values, and likelihood ratios—were calculated for each modality. Inter-reviewer agreement was assessed using Fleiss’ kappa. Our results demonstrated that advanced imaging techniques improved diagnostic performance and inter-reviewer agreement compared to traditional radiography. While specificity (>88%) was comparable to other small animal studies, the sensitivity did not exceed 50% across all modalities. This low sensitivity reflects the challenges posed by minimal fracture displacement and hummingbirds’ extremely small size. Only 3D images achieved high positive likelihood ratios and superior inter-reviewer agreement, highlighting the unique value of 3D visualization in complex anatomical evaluations. Overall, the minute structures of hummingbirds present inherent diagnostic limitations, underscoring that negative radiographic results must be interpreted cautiously, and the possibility of false negatives should prompt consideration of advanced or follow-up imaging when clinical suspicion persists.

## Simple Summary

Hummingbirds are among the smallest birds in the world, and their delicate skeletons make it very difficult for veterinarians to identify fractured bones. This study set out to test which imaging methods are most useful for detecting fractures in ruby-throated hummingbirds brought to a wildlife hospital. Sixteen hummingbirds that had died in the hospital were examined with four techniques: standard X-rays, dental X-rays, micro-computed tomography (micro-CT), and three-dimensional images created from micro-CT scans. The study found that advanced imaging methods, especially three-dimensional reconstructions, improved the ability of veterinarians to detect fractures and increased consistency between reviewers. However, even with these tools, very small fractures were often missed because of the minute size of birds. These findings show that while newer imaging methods can improve care for injured hummingbirds, there will always be limits to what can be seen with current technology.

## 1. Introduction

Wildlife centers and zoos manage a diverse range of species, from small vertebrates like the New Guinea Amau frog (*Paedophryne amauensis*) to towering animals like reticulated giraffes (*Giraffa reticulata*). This diversity requires veterinarians to not only understand in-depth differences in anatomical and physiological characteristics between species but also to adapt diagnostic tools to suit each species. The smallest avian patients admitted to the Wildlife Hospital of Louisiana (Baton Rouge, LA, USA) are ruby-throated hummingbirds (*Archilochus colubris*). Adults only weigh 2–3 g, measure approximately 7–9 cm in body length—comparable to that of a cicada—and have a maximum wingspan of about 10 cm. In such a minute species, physical examination alone is often insufficient for diagnosing fractures. Due to their size, injuries may not be externally visible or palpable unless severely displaced, and manipulation during examination risks worsening trauma. Therefore, imaging plays a critical role in accurate diagnosis while minimizing harm.

Traditional radiography—the most common and widely accepted screening modality in veterinary practice—often proves inadequate for birds of this scale. The main challenges of using equipment designed for larger small animals like dogs and cats include excessive radiation exposure, image over-penetration, and insufficient spatial resolution to detect fine anatomical details [1,2]. Dental radiography—originally designed to assess the oral structures of mammals—has occasionally been employed to image these small animals [3,4,5]. Its compact sensor and relatively low radiation output allow clinicians to position the film as a miniaturized radiography table to capture images of the body or extremities in small species. However, it still falls short due to its narrow field of view, limited penetration power, and resolution [3]. Computed tomography (CT) has become widely used in veterinary medicine, providing cross-sectional views that circumvent the issue of superimposition common in radiography. Yet, the resolution and slice thickness parameters of standard CT systems—optimized for larger small animals—remain suboptimal for birds weighing only a few grams. Since the full body length of a ruby-throated hummingbird is only about 3 cm, conventional CT can acquire only a limited number of slices with insufficient spatial resolution, which markedly reduces its diagnostic value.

In contrast, micro-CT—adopted in laboratory animal research first to accommodate rodents—offers substantially higher spatial resolution and has become a valuable tool for ex vivo and in vivo imaging in very small animals [6,7,8]. This technique, which typically uses a cone-beam geometry, was initially limited to postmortem or excised specimens due to the high radiation doses required [9]. However, recent advancements have enabled low-dose in vivo imaging, expanding its clinical feasibility in minute species. This technique has proven particularly useful for identifying complex or subtle long-bone fractures, like conventional CT does [10]. Furthermore, three-dimensional (3D) reconstructions of CT datasets allow interactive visualization of anatomy from any angle, facilitating more accurate diagnosis, surgical planning, and communication with other professionals.

Despite the increasing availability of imaging modalities, comparative studies on their diagnostic performance in minute avian species remain scarce. Given the practical and ethical considerations involved in imaging such delicate patients, selecting the optimal modality is critical to balance diagnostic accuracy and patient safety. Therefore, our study aimed to compare four techniques—general radiography, dental radiography, micro-CT, and 3D-reconstructed images of micro-CT datasets—for their effectiveness in detecting bone fractures in hummingbirds. We hypothesized that diagnostic performance would improve in the following order: general radiography, dental radiography, micro-CT, and 3D images, with 3D images yielding the highest diagnostic accuracy and inter-reviewer agreement.

## 2. Materials and Methods

### 2.1. Animals

Sixteen ruby-throated hummingbirds admitted to the Wildlife Hospital of Louisiana over an 18-month period (2023–2024) were included in the study. All hummingbirds were euthanized or succumbed to their injuries, including cat attacks and window collisions. These birds were used for this comparative study to minimize the risk of repeated anesthesia in live patients during imaging. At the time of recruitment, the presence or absence of bone fractures in these cases was not known; however, it was suspected that fractures were possibly present in some of the cases and absent in others. The cadavers were frozen (−20 °C) until being imaged, and thawed in a refrigerator one day before the imaging. All three imaging methods (general radiography, dental radiography, micro-CT) were performed on the same day.

### 2.2. Methods

Each bird was imaged using all four modalities, resulting in a total of 64 image sets. General radiographs were obtained using a digital radiography system (DX-D 600; AGFA HealthCare Corp., Greenville, SC, USA) at 50 kV and 5.1 mAs (640 mA, 8 ms exposure time). Dental radiographs were acquired using a dental X-ray unit (Preva; Midmark, Versailles, OH, USA) at 65 kV and 0.056 mAs (7 mA, 8 ms exposure time). Every bird was positioned in the ventrodorsal and right lateral recumbency to obtain two orthogonal views. CT scans were performed without contrast enhancement using a cone-beam micro-CT (Triumph^®^ II; TriFoil Imaging, Chatsworth, CA, USA) with the following parameters: tube voltage of 75 kV, tube current of 110 μA, and exposure time of 230 ms per projection. A total of 512 projections were obtained. The detector was configured to 2 × 2 binning, resulting in an effective pixel size of 0.1 mm, with a magnification factor of 2.0× and a field of view of 59.2 mm. DICOM files obtained from the micro-CT were imported into 3D Slicer software (v.5.8.1; www.slicer.org) for generating 3D reconstructions in an STL format [11]. The final reconstructed images had an isotropic voxel size of 0.1 mm.

All acquired images were uploaded to a picture archiving and communication system (PACS, AGFA HealthCare Corp.) and evaluated on the same medical-grade monitor located in the Louisiana State University Veterinary Teaching Hospital radiology reading room. For CT, only transverse plane images were available for review. Every PACS-uploaded image could be adjusted by each evaluator. The 3D-reconstructed images could be rotated and increased/decreased in size per the reviewer’s desire. The reviewing order for each modality was randomized using a random number generator (www.random.org) by the first author, who was not involved in the image review process. Since the patient number could not be blinded within the PACS, reviewers evaluated one modality per day to reduce recall bias.

Six veterinarians participated in imaging evaluation: two board-certified veterinary radiologists (RJ, KW), one veterinary radiology resident (DO), two wildlife medicine residents (KB, ZL), and one wildlife medicine faculty (KLB). The reviewers were not provided any specific history on the birds and thus were not guided to screen for specific lesions. Reviewers were instructed to report the presence or absence of bone fractures (excluding the skull) and the location of the injury using the four methods (radiographs, dental radiographs, micro-CT transverse scans, and 3D-reconstructed images).

To establish a gold standard for fracture identification, the cadavers were individually introduced into a dermestid beetle (*Dermestes maculatus*) colony for 24 h to remove soft tissues. The first author then confirmed the final diagnosis for each cadaver, as the entire skeleton was exposed without separating any bones.

### 2.3. Statistical Analysis

Diagnostic performance metrics, including sensitivity, specificity, positive predictive value (PPV, precision), negative predictive value (NPV), positive likelihood ratios (LR+), and negative likelihood ratios (LR−), were calculated from contingency tables using Microsoft Excel (v.2019; Microsoft Corp., Redmond, WA, USA) and GraphPad Prism (v.9; San Diego, CA, USA). Inter-reviewer agreement was assessed using Fleiss’ kappa (SPSS v.26; IBM, Armonk, NY, USA). The kappa test was also used to evaluate agreement between faculty and house officers and specialties.

For performance evaluation, the main analysis was based on pooled reviewers’ assessments. Comparison between faculty and house officers, and specialties is also analyzed by pooled methods. A supportive analysis using a majority vote approach (≥4 of 6 reviewers in agreement) was also performed to reflect a practical consensus setting. Cochran’s Q test was used to compare the sensitivity and specificity across the four modalities, using the individual reviewer’s assessments for each case as paired binary outcomes. A *p*-value < 0.05 was considered statistically significant across all analyses.

## 3. Results

**Figure 1:**
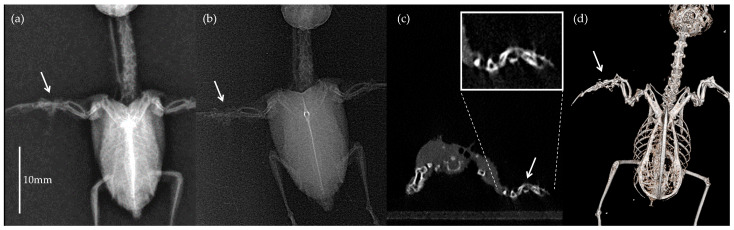
Comparative images of the same ruby-throated hummingbird with a right major and minor metacarpal fractures acquired using four different modalities: (**a**) general radiography, (**b**) dental radiography, (**c**) micro-computed tomography (micro-CT) transverse scan, and (**d**) 3D reconstructed image. The fracture site is identified in each image with an arrow for illustrative purposes; these were not marked during reviewer assessment. A precise scale bar is only provided in (**a**) due to variations in magnification and the nature of the reconstructed data.

Of the 16 birds, 9 (56.3%) had fractures with or without luxations in their wings or bodies. Four birds had multiple fractures. The types of injuries were as follows: scapulohumeral compression fracture (*n* = 2), clavicle fracture (*n* = 2), metacarpal fracture (*n* = 4), keel fracture (*n* = 3), and rib fractures (*n* = 3). Examples of the four different imaging modalities from the same hummingbird with a unilateral metacarpal fracture are shown in Figure 1.

Diagnostic performance metrics based on pooled reviewers’ assessments are presented in Table 1. As the imaging modality advanced, all diagnostic indices generally increased. Notably, only 3D-reconstructed images had a positive likelihood ratio (LR+) above 10 [12]. None of the negative likelihood ratios (LR−) were below 0.5. All Fleiss’ kappa values (0.405–0.576) fell into a moderate level of agreement among reviewers [13], with the highest being measured for 3D images, followed by micro-CT, dental radiography, and general radiography, in that order. Using the reviewers’ individual case-level assessments, Cochran’s Q test revealed no significant differences in sensitivity or specificity among the imaging modalities (all *p* > 0.5).

In the supportive analysis using the majority vote approach (≥4 of 6 reviewers), general radiography still showed the poorest performance based on sensitivity and NPV, while the other three methods had the same, slightly higher metrics (Table A1). Notably, all methods had 100% specificity and positive predictive value. As no false positives were identified, LR+ was infinite across all methods, while LR– values consistently remained above 0.5.

The performance metrics calculated by faculty and resident groups are shown in Table 2. Sensitivity was higher in the resident group (40.7–55.6%) than in the faculty group (33.3–44.4%), while specificity was consistently higher in the faculty (95.2–100.0%) than in the residents (81.0–90.5%). Positive predictive values were also higher in faculty (90.0–100.0%) relative to residents (73.3–88.2%), whereas negative predictive values were similar between groups. Fleiss’ kappa values indicated moderate agreement among faculty reviewers (0.495–0.667); however, agreement among residents was generally lower (0.250–0.438). When reviewers were grouped by specialty (Table 3), radiology-trained veterinarians showed higher sensitivity than those trained in wildlife medicine for micro-CT transverse images (52% vs. 33%); while sensitivity for 3D-rendered images was nearly identical between specialties (50% vs. 48%).

## 4. Discussion

Our results support the initial hypothesis, demonstrating that advanced imaging techniques generally improve diagnostic performance and inter-reviewer agreement compared to traditional general radiography. However, the performance metrics we observed here were not as high as those typically seen in dogs and cats, which have a median sensitivity of 86% and an LR+ > 13 [14]. This discrepancy reflects the inherent difficulties of evaluating such diminutive birds with our current methods. Specificity in our study was comparable to that of small animals, but sensitivity was only about half as high. This was confirmed by the high PPV (80–93%), indicating that fractures identified were likely true positives. In contrast, the lower NPV (53–60%) reflected the main diagnostic challenge of detecting all existing injuries. The higher false negative rate was unsurprising, attributed to the hummingbird’s diminutive size and the minimal displacement, which often restricts the ability to visually discern the fractures. Consistently elevated LR− values (>0.5) further highlight this limited rule-out utility of the tested modalities in this species. Notably, when decisions were based on a majority vote consensus among reviewers, the scoring was more conservative, resulting in perfect specificity and NPV (100%). This emphasizes that consensus scoring can reduce false positives, though it may further limit sensitivity in detecting subtle fractures, underscoring the trade-off between diagnostic confidence and detection capability [15].

The challenge with imaging hummingbirds was particularly evident in the case of general radiography, which had the lowest diagnostic performance. Even with post-adjustment of brightness and contrast in PACS by users, overexposure and limited resolution could not be adequately corrected for hummingbirds, unless they had an easily visible keel fracture or displacement. Moreover, targeted views and orthogonal projections for suspected ranges of interest were not applicable due to their small sizes, limiting manipulation of their body parts and fundamental resolution limitations, which could have increased the diagnostic performance in general. We used an intermediate energy setting, which is typically chosen for balancing soft tissue and bone contrast in clinical practice. Slightly lowering the kVp to enhance bone contrast and increasing the mAs value to reduce quantum noise, if the animal is stationary, might yield marginally better resolution [16,17]. However, longer exposure time in anesthetized patients carries the risk of motion blur due to rapid respiration, further deteriorating visibility [18,19]. In the past, mammography film was used for birds weighing less than 100 g to achieve high resolution [19,20], but it has largely been replaced by digital systems due to its higher radiation exposure requirements and longer exposure time, which induces motion artifacts [21,22,23]. Dental radiography partially compensated for overexposure issues due to its lower radiation intensity, and the skeletal borders were more clearly delineated than in general radiography. Nonetheless, it still demonstrated the same limitations due to the summated structures and the fundamentally minute degree of displacement, resulting in the second-lowest metrics.

As predicted, micro-CT and its 3D-reconstructed skeletons outperformed other modalities, but even these did not reach the diagnostic levels typically seen in small animals. One possible reason was that reviewers could only evaluate transverse views since the images were not obtained on a CT system integrated with PACS, which prevented multiplanar reconstruction for sagittal and coronal scans. This meant that subtle fractures with minimal displacement could be easily overlooked when relying on a single plane. A combined evaluation of all three planes would likely lead to an even greater increase in sensitivity. Although micro-CT did not dramatically exceed the performance of other two-dimensional modalities, it still provided clear advantages by eliminating superimposition and offering cross-sectional detail. Increased inter-rater agreement was consistent with findings reported in a study comparing radiography and CT for the evaluation of the canine pelvis [24]. While the authors have reported that inter-reviewer discrepancies are most prominent in specific fracture locations, we did not have a sufficient number of samples to conduct a similar categorical analysis based on fracture location.

Three-dimensional reconstructed images demonstrated a marked improvement in all diagnostic metrics. Sensitivity remained modest at 50% (the highest among modalities), but specificity reached 95%, and the LR+ exceeded 10—values strongly supporting their diagnostic and clinical utility [12]. While micro-CT transverse scans provided superior detail without superimposition, the 3D-rendered images offered an intuitive and readily interpretable view. This allowed reviewers to more consistently assess fracture location, type, and displacement. In particular, this was effective for visualizing dislocation without overt cortical bone breakage, as it enabled manipulation in a 360-degree view and direct comparison with the contralateral side. Inter-reviewer agreement was also highest at 70%, further supporting that this modality provided reviewers with the most confidence. The clinical application of reconstructed cone-beam CT images has also been shown to be useful in evaluating maxillofacial fractures of cats [25].

Although 3D reconstructions are not a standard method for CT interpretation, they were preferred by clinicians who were not specialized in radiology. Notably, the gap in sensitivity observed between radiologists and non-radiologists with micro-CT was minimized when evaluating 3D reconstructed images, suggesting practical value in general clinical settings. This finding indicates that while advanced training enhances interpretation of traditional CT slices, 3D visualization helps level the field, making fracture detection more accessible across varying backgrounds.

Indeed, reviewer expertise also influenced performance as shown in another study conducted in dogs [26,27,28]. Higher sensitivity in the resident group and higher specificity in the faculty group found in our study indicate that residents favored detecting more fractures even at the cost of more false positives, while faculty prioritized being conservative, achieving higher specificity (>95%). The reduced agreement among residents likely reflects greater variability in individual reviewer performance. Nonetheless, performance did not always correlate with experience, highlighting the importance of consensus and communication.

A few limitations of our study design should be considered when interpreting our findings. Firstly, it is essential to contextualize the micro-CT system’s performance. The utilized protocol was specifically optimized for low-dose, in vivo imaging to meet ethical requirements for live animal assessment, resulting in an effective pixel size of 0.1 mm. We acknowledge that this chosen resolution does not represent the maximal spatial performance of high-dose, ex vivo micro-CT typically employed for post-mortem anatomical studies. Consequently, the observed diagnostic metrics support comparisons based on clinically and ethically achievable resolution, rather than the theoretical maximum of the technology. Furthermore, the use of postmortem specimens, while ethically necessary, eliminates key variables such as patient motion and the opacity and density variations in soft tissue. These facts present significant practical challenges in a clinical setting and would likely affect the visibility of in vivo images. Third, the small sample size and lack of standardized fracture types limit the generalizability of our findings. Moreover, none of the fractures in these cases were in proximal large long bones. These cases were opportunistically sampled from all cases available, and thus, the types of injuries could not be controlled. However, we believe that these distributions reflect clinically relevant representation of the common injuries of this species in our region. Next, all reviewers had less than five years of post-graduate experience in their field, which could have influenced the results. Additionally, although thorough physical examination is often limited in hummingbirds, reviewers in clinical settings usually receive some physical examination findings or clinical history, which help them focus on relevant areas during image interpretation. Because no such information was available in our study, there is a chance that their evaluations were less targeted than they would be in a real clinical scenario. Finally, our study did not consider the time and cost required for image acquisition or review, which are crucial factors in a clinical practice. Thus, while 3D rendered images using micro-CT scans demonstrated the highest diagnostic value, their clinical utility may be constrained by limited accessibility and higher costs compared to conventional radiography.

Despite the limitations, our findings provide critical guidance for small avian caretakers. Conventional or dental radiography remains a reasonable first-line screening tool given its accessibility and low cost, but our low sensitivity results strongly suggest that negative radiographic findings must not be relied upon to exclusively rule out a fracture. The superior diagnostic metrics and reduced subjectivity achieved by 3D-reconstructed images establish them as the most accurate method for definitive fracture diagnosis. However, we acknowledge that the high cost, specialized training requirements, and limited accessibility of micro-CT equipment present significant barriers to routine clinical translation. Ultimately, our study suggests that the decision to pursue advanced imaging should be based on a judicious balance between clinical urgency, resource availability, and the potential impact of a missed diagnosis on patient outcome.

## 5. Conclusions

Our diagnostic findings, derived using a veterinary radiography; dental radiography; and low-dose, in vivo-capable micro-CT protocol, highlight that the primary diagnostic challenge for those working with hummingbirds is the high false negative rate across all modalities. This emphasizes that clinicians should be cautious about ruling out fractures solely based on negative findings and should consider multimodal approaches, repeat imaging, or clinical follow-up when suspicion remains high. In practice, advanced imaging—particularly 3D reconstructions—can improve diagnostic confidence and inter-reviewer agreement.

## Figures and Tables

**Table 1:** Diagnostic performances and agreement among all six reviewers of each imaging modality (%, except for likelihood ratios and kappa values) with 95% confidence intervals in brackets.

Modality	Sensitivity	Specificity	PPV	NPV	LR+	LR−	k
General radiography	37.0 (25.4–50.4)	88.1 (75.0–94.8)	80.0 (60.9–91.1)	52.1 (40.7–63.3)	3.1 (1.3–7.6)	0.7 (0.6–0.9)	0.405 (0.401–0.409)
Dental radiography	40.7 (28.7–54.0)	88.1 (75.0–94.8)	81.5 (63.3–91.8)	53.6 (42.0–64.9)	3.4 (1.4–8.3)	0.7 (0.5–0.9)	0.433 (0.429–0.437)
Micro-CT	42.6 (30.3–55.8)	88.1 (75.0–94.8)	82.1 (64.4–92.1)	54.4 (42.7–65.7)	3.6 (1.5–8.6)	0.7 (0.5–0.8)	0.536 (0.532–0.540)
3D-reconstructed image	50.0 (37.1–62.9)	95.2 (84.2–98.7)	93.1 (78.0–98.1)	59.7 (47.7–70.6)	10.4 (2.7–41.7)	0.5 (0.4–0.7)	0.576 (0.572–0.581)

Abbreviations: CT: computed tomography; PPV: positive predictive value; NPV: negative predictive value; LR+: positive likelihood ratios; LR−: negative likelihood ratios; k: kappa statistic.

**Table 2:** Diagnostic performances and agreement of each imaging modality by their experience level (%, except for kappa values).

Modality	Reviewer Group	Sensitivity	Specificity	PPV	NPV	k
General radiography	Faculty	33.3%	95.2%	90.0%	52.6%	0.495
	Resident	40.7%	81.0%	73.3%	51.5%	0.321
Dental radiography	Faculty	37.0%	95.2%	90.9%	54.1%	0.646
	Resident	44.4%	81.0%	75.0%	53.1%	0.250
Micro-CT	Faculty	37.0%	95.2%	90.9%	54.1%	0.646
	Resident	48.1%	81.0%	76.5%	54.8%	0.362
3D-reconstructed image	Faculty	44.4%	100.0%	100.0%	58.3%	0.667
	Resident	55.6%	90.5%	88.2%	61.3%	0.438

Abbreviations: CT: computed tomography; PPV: positive predictive value; NPV: negative predictive value; k: kappa statistic.

**Table 3:** Diagnostic performances and agreement of each imaging modality by their training specialty (%, except for kappa values).

Modality	Reviewer Group	Sensitivity	Specificity	PPV	NPV	k
General radiography	Wildlife medicine	40.7	90.5	84.6	54.3	0.578
	Radiology	33.3	85.7	75.0	50.0	0.222
Dental radiography	Wildlife medicine	33.3	90.5	81.8	51.4	0.292
	Radiology	48.1	85.7	81.3	56.3	0.437
Micro-CT	Wildlife medicine	33.3	95.2	90.0	52.6	0.621
	Radiology	51.8	81.0	77.8	56.7	0.467
3D-reconstructed image	Wildlife medicine	48.1	100.0	100.0	60.0	0.684
	Radiology	49.6	90.5	87.5	59.4	0.515

Abbreviations: CT: computed tomography; PPV: positive predictive value; NPV: negative predictive value; k: kappa statistic.

## Data Availability

The original datasets presented in the study are included in the article/Appendix A; further inquiries can be directed to the corresponding author.

## Appendix A

**Table A1:** Diagnostic performance of each modality (%, except for likelihood ratios), determined by majority vote (≥4 reviewers out of 6) decision with 95% confidence intervals in brackets.

Modality	Sensitivity	Specificity	PPV	NPV	LR+	LR-
General radiography	33.3 (12.1–64.6)	100 (64.6–100)	100 (43.9–100)	53.8 (29.1–76.8)	Infinity	0.6 (0.3–1.0)
Dental radiography	44.4 (18.9–73.3)	100 (64.6–100)	100 (51.0–100)	58.3 (32.0–80.7)	Infinity	0.7 (0.4–1.1)
Micro-CT	44.4 (18.9–73.3)	100 (64.6–100)	100 (51.0–100)	58.3 (32.0–80.7)	Infinity	0.6 (0.3–1.0)
3D-reconstructed image	44.4 (18.9–73.3)	100 (64.6–100)	100 (51.0–100)	58.3 (32.0–80.7)	Infinity	0.6 (0.3–1.0)

Abbreviations: CT: computed tomography; PPV: positive predictive value; NPV: negative predictive value; LR+: positive likelihood ratios; LR−: negative likelihood ratios; k: kappa statistic.

## References

[1] Vilaplana Grosso F. (2019). Orthopedic Diagnostic Imaging in Exotic Pets. Vet. Clin. Exot. Anim. Pract..

[2] Dennison S.E., Speer B., Zeeland Y.R.A.v. (2025). Common Imaging Modalities: Selecting the Right Modality and Getting the Most out of Your Imaging. Current Therapy in Avian Medicine and Surgery.

[3] Silverman S., Tell L.A. (2010). Radiology of Birds: An Atlas of Normal Anatomy and Positioning.

[4] Nugent-Deal J. Radiographic Techniques and Positioning of Exotic Companion Animals. Proceedings of the Atlantic Coast Veterinary Conference.

[5] Capello V., Lennox A.M. (2008). Clinical Radiology of Exotic Companion Mammals.

[6] Arai Y., Yamada A., Ninomiya T., Kato T., Masuda Y. (2005). Micro-Computed Tomography Newly Developed for in Vivo Small Animal Imaging. Oral Radiol..

[7] Li H., Zhang H., Tang Z., Hu G. (2008). Micro-Computed Tomography for Small Animal Imaging: Technological Details. Prog. Nat. Sci..

[8] Clark D.P., Badea C.T. (2021). Advances in Micro-Ct Imaging of Small Animals. Phys. Medica.

[9] Willekens I., Buls N., Lahoutte T., Baeyens L., Vanhove C., Caveliers V., Deklerck R., Bossuyt A., de Mey J. (2010). Evaluation of the Radiation Dose in Micro-CT with Optimization of the Scan Protocol. Contrast Media Mol. Imaging.

[10] Sasai H., Fujita D., Tagami Y., Seto E., Denda Y., Hamakita H., Ichihashi T., Okamura K., Furuya M., Tani H. (2015). Characteristics of Bone Fractures and Usefulness of Micro–Computed Tomography for Fracture Detection in Rabbits: 210 Cases (2007–2013). J. Am. Vet. Med. Assoc..

[11] Fedorov A., Beichel R., Kalpathy-Cramer J., Finet J., Fillion-Robin J.-C., Pujol S., Bauer C., Jennings D., Fennessy F., Sonka M. (2012). 3D Slicer as an Image Computing Platform for the Quantitative Imaging Network. Magn. Reson. Imaging.

[12] Jaeschke R., Guyatt G.H., Sackett D.L., Guyatt G., Bass E., Brill-Edwards P., Browman G., Cook D., Farkouh M., Gerstein H. (1994). Users’ Guides to the Medical Literature: III. How to Use an Article About a Diagnostic Test B. What Are the Results and Will They Help Me in Caring for My Patients?. J. Am. Med. Assoc..

[13] Landis J.R., Koch G.G. (1977). The Measurement of Observer Agreement for Categorical Data. Biometrics.

[14] Lamb C.R., Nelson J.R. (2015). Diagnostic Accuracy of Tests Based on Radiologic Measurements of Dogs and Cats: A Systematic Review. Vet. Radiol. Ultrasound.

[15] Scrivani P.V. (2002). Assessing Diagnostic Accuracy in Veterinary Imaging. Vet. Radiol. Ultrasound.

[16] Thrall D.E. (2012). Textbook of Veterinary Diagnostic Radiology.

[17] Muhlbauer M.C., Kneller S.K. (2024). Radiography of the Dog and Cat: Guide to Making and Interpreting Radiographs.

[18] Helmer P., Harrison G., Lightfoot T. (2006). Advances in Diagnostic Imaging. Clinical Avian Medicine.

[19] Krautwald-Junghanns M.E., Schroff S., Bartels T., Krautwald-Junghanns M.E., Pees M., Reese S., Tully T. (2011). Radiographic Investigation. Diagnostic Imaging of Exotic Pets: Birds, Small Mammals, Reptiles.

[20] Ketz-Riley C.J., Sanchez C.R., Miller R.E., Fowler M.E. (2014). Trochiliformes (Hummingbirds). Fowler’s Zoo and Wild Animal Medicine.

[21] McMillan M., Ritchie B., Harrison G., Harrison L. (1995). Imaging Techniques. Avian Medicine: Principles and Application.

[22] Krautwald-Junghanns M.E., Tully T.N., Dorrestein G.M., Jones A.K. (2009). Imaging Techniques. Handbook of Avian Medicine.

[23] Bochmann M., Ludewig E., Krautwald-Junghanns M.E., Pees M. (2011). Comparison of the Image Quality of a High-Resolution Screen–Film System and a Digital Flat Panel Detector System in Avian Radiography. Vet. Radiol. Ultrasound.

[24] Crawford J.T., Manley P.A., Adams W.M. (2003). Comparison of Computed Tomography, Tangential View Radiography, and Conventional Radiography in Evaluation of Canine Pelvic Trauma. Vet. Radiol. Ultrasound.

[25] Heney C.M., Arzi B., Kass P.H., Hatcher D.C., Verstraete F.J.M. (2019). Diagnostic Yield of Dental Radiography and Cone-Beam Computed Tomography for the Identification of Anatomic Structures in Cats. Front. Vet. Sci..

[26] Hansson K., Häggström J., Kvart C., Lord P. (2009). Reader Performance in Radiographic Diagnosis of Signs of Mitral Regurgitation in Cavalier King Charles Spaniels. J. Small Anim. Pract..

[27] Alexander K., Joly H., Blond L., D’Anjou M.-A., Nadeau M.-È., Olive J., Beauchamp G. (2012). A Comparison of Computed Tomography, Computed Radiography, and Film-Screen Radiography for the Detection of Canine Pulmonary Nodules. Vet. Radiol. Ultrasound.

[28] Stieger-Vanegas S.M., Senthirajah S.K.J., Nemanic S., Baltzer W., Warnock J., Bobe G. (2015). Evaluation of the Diagnostic Accuracy of Four-View Radiography and Conventional Computed Tomography Analysing Sacral and Pelvic Fractures in Dogs. Vet. Comp. Orthop. Traumatol..

